# Astrocytes expressing mutant hnRNPA1 induce non-cell-autonomous motor neuron death

**DOI:** 10.1016/j.brainresbull.2025.111522

**Published:** 2025-08-24

**Authors:** Qinxue Wu, Xionghao Liu, Tingting Zhang, Shiquan Cui, Bo Huang, Cao Huang, Qilin Cao, Xu-Gang Xia, Hongxia Zhou

**Affiliations:** a Department of Pathology, Thomas Jefferson University, 1020 Locust Street, Philadelphia, PA 19107, USA; b Department of Environmental Health Sciences, Robert Stempel College of Public Health & Social Work, Florida International University, FL 34987, USA

**Keywords:** HnRNPA1, ALS, Transgenic rats, Astrocytes, Non-cell autonomous neuron death

## Abstract

Pathogenic mutation of heterogeneous nuclear ribonucleoprotein A1 (hnRNPA1) is causative to amyotrophic lateral sclerosis (ALS). Neuron death resulting from pathogenic hnRNPA1 may not require its presence across all pertinent cells types, including neurons, glia, and muscles. Rather, the exclusive presence of pathogenic hnRNPA1 in a specific cell type, such as astrocytes, may suffice to substantially alter cellular functions. Consequently, this alteration initiates abnormal interaction within intricate neuron-glia networks, culminating in non-cell-autonomous motor neuron death. To investigate the pivotal role of non-cell-autonomous neuron death in hnRNPA1-associated ALS, we developed transgenic rats overexpressing mutant hnRNPA1 in specifically astrocytes. The confined overexpression of pathogenic hnRNPA1 in astrocytes instigated a sequence of events resulting in motor neuron death and subsequent muscle atrophy. These findings underscore the critical, non-cell-autonomous contribution of astrocytes to hnRNPA1-induced neurodegeneration in ALS, and point toward astrocytic pathways as potential therapeutic targets.

## 1. Introduction

Amyotrophic lateral sclerosis (ALS) results from progressive motor neuron degeneration, ultimately leading to muscle weakness and paralysis (Ilieva et al., 2009; Gendron et al., 2014). While most ALS cases are sporadic, the familial form of ALS can arise from genetic mutations (Deng et al., 1993; Rosen et al., 1993; Gurney et al., 1994; Vance et al., 2009; Kwiatkowski et al., 2009; Kabashi et al., 2008; Sreedharan et al., 2008; Van Deerlin et al., 2008; Rutherford et al., 2008; Kim et al., 2013). Genetic mutations, such as those in the *SOD1, C9orf72, TDP-43, FUS*, and *UBQLN2* genes, contribute to familial ALS cases. Understanding the genetic basis of ALS is crucial for elucidating neurodegenerative pathways, identifying effective biomarkers for monitoring disease progression, and assessing therapeutic intervention. The exact mechanism of motor neuron degeneration in ALS is complex and multifaceted. Aberrant protein aggregation, mitochondrial dysfunction, excitotoxicity, and impaired RNA metabolism are among the key molecular events implicated in ALS pathogenesis. Glutamate excitotoxicity, oxidative stress, and disrupted axonal transport further contribute to the selective vulnerability of motor neurons. The interplay of these factors results in a cascade of events leading to motor neuron degeneration in the disease, highlighting the need for comprehensive therapeutic strategies targeting various aspects of the disease pathways.

Notably, pathogenic mutation in heterogeneous nuclear ribonucleoprotein A1 (hnRNPA1) has been identified in the families afflicted with ALS (Kim et al., 2013). HnRNPA1 is a component of heterogeneous ribonucleoprotein particles, primarily localized in the nucleoplasm. It serves as a platform for mRNA processing, including splicing, packaging, and nuclear exportation (Jean-Philippe et al., 2013; Gagné et al., 2021). HnRNPA1 plays a role in regulating SMN2 mRNA splicing by inhibiting exon-7 inclusion and suppressing cryptic exon inclusion during ATM mRNA splicing (Pastor and Pagani, 2011; Kashima et al., 2007). Additionally, hnRNPA1 interacts with IκBα to facilitate IκBα degradation, activating NF-κB and thereby regulating its transcriptional activity (Hay et al., 2001). Post-translational modification, such as methylation, influences hnRNPA1 function. Methylation, catalyzed by specific methyltransferases, reduces the RNA binding capacity of hnRNPA1 and possibly facilitates its nuclear export (Kim et al., 1998; Shen et al., 1998). ZMYND11 specifically recognizes hnRNPA1 methylated at arginine 194 and sequesters it within the nucleus, thereby inhibiting its translocation to the cytoplasm and preventing stress granule formation (Lian et al., 2024). Clinical studies indicate that different hnRNPA1 mutations cause varying clinical manifestations (Kim et al., 2013).

Creating a reliable model system is crucial for understanding hnRNPA1 pathobiology. While cellular models offer insights into certain disease aspects, an animal model is essential for unraveling complex neurodegeneration mechanisms. Existing evidence from SOD1 transgenic mice highlights the significance of animal models in studying ALS-related processes, such as axonal transport and glutamate toxicity (Beal, 2001; Menzies et al., 2002; Rothstein, 1996; Ikonomidou et al., 1996; Bittigau and Ikonomidou, 1997; Zhang et al., 1997; Williamson and Cleveland, 1999). These animal models have provided valuable insights into the progressive degeneration of motor neurons and the accompanying motor deficits characteristic of ALS. Studies in animal models have also explored novel treatment strategies, including gene therapies, small molecules, and stem cell-based approaches, aiming to mitigate disease progression and enhance motor function. Despite identifying SOD1 mutations in ALS patients several decades ago, the molecular basis of the toxic gain-of-function phenotype and the mechanism of neuron death remain incompletely understood (Cleveland and Rothstein, 2001; Brown and Robberecht, 2001; Siddique and Lalani, 2002). This underscores the necessity for more sophisticated animal models, particularly for hnRNPA1 research, to reproduce the complex phenotypes associated with hnRNPA1 mutations in a mammalian context.

The induction of neurodegeneration may not necessitate the pathogenic alteration of hnRNPA1 in all relevant cell types, including neurons, glia, and muscles. The presence of pathogenic hnRNPA1 in a specific cell type such as astrocyte may suffice to significantly alter cellular functions and initiate abnormal interaction within intricate neuron-glia networks, leading to non-cell-autonomous neuron death. Neuronal damage and dysfunction are not solely governed by intrinsic factors within the affected neurons, but are also influenced by interactions with neighboring cells and the broader cellular environment. This intricate process involves intricate crosstalk between neurons, glial cells, and other non-neuronal elements. In various neurodegenerative diseases, such as Alzheimer’s, Parkinson’s, and ALS, non-cell-autonomous mechanisms play a crucial role in disease progression. Factors like inflammatory responses, impaired glial support, and the release of toxic molecules from neighboring cells contribute to the death of neurons. Along with hnRNPA1, TDP-43 and FUS are heterogeneous ribonucleoproteins with similar molecular roles (Kwiatkowski et al., 2009; Tong et al., 2013; Huang et al., 2012). Previous research has demonstrated that the constrained overexpression of pathogenic TDP-43 in motor neurons or astrocytes is adequate to induce cell-autonomous and non-cell-autonomous motor neuron death, respectively (Tong et al., 2013; Huang et al., 2012). Moreover, research involving the restricted expression of mutant SOD1 in specific cell types has provided crucial insights into the selective vulverability observed in motor neuron disease (Yamanaka et al., 2008; Pramatarova et al., 2001). However, distinct genetic mutations may lead to unique patterns of neuron degeneration. For instance, Purkinje neurons undergo cell-autonomous degeneration in response to *NPC1* gene mutation (Ko et al., 2005), while they exhibit non-cell-autonomous degeneration in response to *ataxia-7* gene mutation (Custer et al., 2006). The specific role of hnRNPA1 in the cellular mechanisms of ALS, particularly in non-neuronal cells such as astrocytes, remains to be elucidated. Recent studies suggest that hnRNPA1’s interactions with stress granules, non-coding RNAs, and epigenetic modifiers may influence its pathogenic potential in different cellular contexts.

To investigate hnRNPA1 pathobiology, we developed transgenic rats that overexpressed hnRNPA1 bearing a mutation found in ALS patients. The resultant transgenic rats, specifically overexpressing mutant hnRNPA1 in astrocytes, manifested a progressive paralysis phenotype arising from severe motor neuron demise and subsequent muscle atrophy. These observations indicate that the confined overexpression of pathogenic hnRNPA1 in astrocytes alone is adequate to instigate non-cell-autonomous motor neuron death.

## 2. Materials and methods

### 2.1. Development of hnRNPA1 rat model

The animal studies received approval from the Institutional Animal Care and Use Committees at Thomas Jefferson University (protocol number: 01370) and Florida International University (protocol number: 201157). These studies were conducted in strict adherence to NIH guidelines, as well as local legislation and institutional requirements.

The study was not preregistered and no sample calculation was performed. In this study, 150 rats were used for generating and characterizing TRE-hnRNPA1 transgenic lines and no animal was excluded from the study. No randomization was performed to allocate subjects and no blinding was performed. For characterizing disease phenotypes, each group consisted of 12 rats (6 males and 6 females). For prefixing tissues, rats were deeply anesthetized with ketamine (10 mg/ml)/xylazine (1 mg/ml) mixture and were then perfused with 4 % paraformaldehyde. For terminal tissue collection, rats were deeply anesthetized with IsoFlurane and then were rapidly decapitated prior to tissue dissection. After administration of anesthesia and monitoring of foot and tail withdrawal reflexes, the rats were rapidly decapitated.

Transgenic rats expressing mutant hnRNPA1 were generated through pronuclear injection and maintained on Sprague-Dawley (SD) genomic background, following established protocols (Tong et al., 2013). Rat hnRNPA1 cDNA was reverse-transcribed from total mRNA isolated from SD rat’s brain, and the opening reading frame (ORF) of rat hnRNPA1 was cloned into a transgenic plasmid (Tong et al., 2013). Two splicing isoforms were derived for rat hnRNPA1, with the short isoform being predominant; consequently, the short hnRNPA1 isoform was selected for transgenic study. The ALS-linked D314N mutation was introduced via PCR-based mutagenesis of the rat hnRNPA1 open reading frame (ORF). The mutant ORF was then cloned between the tetracycline-responsive element (TRE) promoter and SV40 poly (A) signaling sequence (Tong et al., 2013). Transgenic rats harboring the TRE-hnRNPA1 construct were identified by PCR using the primers 5’-TTGTTTGTGGATCGCTGTGA-3’ (forward) and 5’-GACAAACTTCACGTCAGGGT-3’ (reverse), and sequencing of the PCR products confirmed the presence of the D314N substitution in transgenic hnRNPA1.Transgenic lines stably transmitting the TRE-hnRNPA1 transgene were selected for phenotypic analysis. The GFAP-tTA transgenic rats, previously characterized (Tong et al., 2013), were utilized. Selected TRE-hnRNPA1 rats were bred with GFAP-tTA transgenic rats to produce double transgenic offspring that would express hnRNPA1 specifically in astrocytes. To suppress transgene expression during development, Doxycycline (Dox) was administered to breeding rats. TRE-hnRNPA1/GFAP-tTA double transgenic rats continued to drink Dox-containing water (50 μg/ml) until 35 days old, ensuring that TRE-hnRNPA1 transgene was maintained inactive during embryonic and postnatal development.

### 2.2. Behavioral analyses

In accordance with established lab protocols (Tong et al., 2013), rat mobility was evaluated using Open Field assay and Rotarod test. The distance traveled by a rat in 10 min was recorded in an open field chamber (27.9 cm × 29.9 cm, Med Associates), while the time a rat maintained on a rotating rod was recorded using an Economex accelerating Rotarod (Columbus Instruments). Both assays determined the age at which a transgenic rat exhibited an unrecoverable decline in Open Field travel distance or Rotarod fall latency, marking the onset of ALS disease phenotypes. Paralysis was defined as leg dragging or the inability to retract individual legs. Disease end-stages were characterized by the inability to retract two or more legs or to right itself when placed on its side. Each behavior test group comprised 12 rats with equal sex distribution, and the same groups were utilized for both Open Field activity tests and Rotarod assessments.

### 2.3. Unbiased neuron counting

Following established protocols (Huang et al., 2012), neurons in tissue sections were visualized using Cresyl violet staining and quantified via stereological cell counting. This method is widely used to accurately estimate the total number of neurons in defined central nervous system regions, such as segments of the spinal cord, by overcoming the biases and inaccuracies inherent in traditional sampling techniques. Stereology involves systematic and random sampling of sections throughout the entire segment of lumbar spinal cord, utilizing three-dimensional principles to ensure an unbiased representation of the studied structures. The Stereologer software, operated on a PC connected to a Nikon 80i microscope with a motorized XYZ stage, estimated the number of targeted neurons. Initially, a low-magnification view outlined the targeting area and established a random sampling grid. Then, at high magnification, the program randomly generated an optical dissector probe in the designated area. Neurons meeting predefined inclusion and exclusion criteria were identified, and this process was replicated across all chosen sections. The software calculated the total number of defined neurons based on random counts. Cross-sections of the L3-L5 lumbar spinal cord, each 30 μm thick, were prepared, and motor neurons with a diameter exceeding 25 μm were counted across the entire lumbar spinal cord sections. The counting scopes were outlined under 4X objective and the neurons meeting the criteria were counted under the 40X objective of microscope. Specifically, the frame area was set at 40 × 40 μm with frame height of 8 μm and the guard height of 1 μm. Every 10th section was stained with Cresyl violet and counted for spinal motor neurons on the both sides of L3-L5 lumbar cords. On average, a total of 16–17 sections were counted for each rat. These counts were then extrapolated volumetrically to estimate the total number of motor neurons in the entire lumbar spinal cord, with an average of approximately 380 motor neurons per section serving as a sampling parameter in the broader estimation. Similarly, neurons in the frontal cortex and dentate gyrus were quantified using previously described methods (Tong et al., 2012). In brief, neurons in the frontal cortex and dentate gyrus on one hemisphere were estimated with stereology cell counting system. The frontal cortex was defined as the cortical area from the rostral of forebrain above the optic chiasm to the appearance of corpus callosum. Every 12th section of 30 μm thickness was stained with Cresyl violet and counted for cortical neurons above the line passing the peak of corpus callosum and vertical to the midline after the appearance of corpus callosum. The frame area was set at 30 × 30 μm with the frame height of 8 μm and the guard height of 1 μm. On average, 16–18 sections were counted for the neurons of frontal cortex on one hemisphere. Similarly, the entire dentate gyrus on one hemisphere was counted for the hippocampal neurons. Every 12th section of 20 μm thickness through the whole dentate gyrus area was stained and on average a total of 16–18 sections were counted for each rat. The frame area was set at 8 × 8 μm with the frame height of 8 μm and the guard height of 0.5 μm.

### 2.4. Muscle histology and histochemistry

As outlined previously (Tong et al., 2013), denervation atrophy in the gastrocnemius muscle was assessed using H&E staining and histochemistry for ATPase and nonspecific esterase (NSE) in transgenic rats. Gastrocnemius muscles from euthanized rats were dissected and placed on a plastic module filled with half-hydrated gum. After incubating in the gum for 1 h at room temperature, the muscles embedded in gum were quickly frozen using dry ice-cooled isopentane. The frozen muscles were then sectioned into 14 μm thickness and mounted onto glass slides.

For hematoxylin and eosin (H&E) staining, muscle sections were initially stained with hematoxylin, rinsed, and differentiated for optimal contrast. Subsequently, eosin staining provided color to cytoplasmic structures. Tissue sections underwent dehydration, clearing with xylene, and were mounted on slides with coverslip. Muscle structures were examined using a light microscope.

ATPase staining was employed to discern and categorize different muscle fiber types based on their adenosine triphosphatase (ATPase) activity in acidic or basic solutions. Muscle sections underwent preincubation at either pH 4.3 or pH 9.4 to deactivate the myosin-ATPase enzyme specific to certain muscle fiber types. Subsequently, they were exposed to a color reaction buffer containing cobalt, which formed a black insoluble compound upon ammonium sulfide precipitation during ATP hydrolysis. ATPase staining revealed three muscle fiber types: Type I (slow-twitch oxidative), Type IIa (fast-twitch oxidative), and Type IIb (fast-twitch glycolytic). The visual examination and documentation of various muscle fiber structures were carried out under a light microscope.

Nonspecific esterase (NSE) staining was employed to detect and pinpoint esterase activity in skeletal muscles, illustrating denervated muscle fibers and neuromuscular junctions. In brief, muscle sections were immersed in a staining solution with alpha-naphthyl acetate, swiftly rinsed in running tap water, dried, dehydrated, and then mounted on coverslips. Muscle fiber structures were observed and documented using a Nikon microscope.

### 2.5. Immunostaining and toluidine staining

Rats were deeply anesthetized, transcardially perfused with chilled phosphate-buffered saline, and followed by 4 % paraformaldehyde. The prefixed spinal cords were dissected and further fixed in the same solution at 4° overnight. After thorough fixation, the tissues were embedded and sectioned using a cryostat. Cross-sections, each 20 μm thin, were obtained from the lumbar spinal cord and immunostained with primary antibodies: antibody against GFAP (RRID: AB_94844), antibody against Iba-1 (RRID: AB_839504), antibody against NeuN (RRID: AB_2298772), and antibody recognizing hnRNPA1 (RRID: AB_2117177). Immunofluorescence staining was observed and documented using a Nikon fluorescence microscope as described previously (Tong et al., 2013).

Motor axons in ventral roots were visualized using toluidine staining, following the procedures outlined previously (Tong et al., 2013). Toluidine blue staining is extensively employed for visualizing nerve axon structures. This metachromatic dye selectively binds to acidic components like nucleic acids and sulfated proteoglycans in tissues. In the study of motor axons, toluidine blue specifically stains myelin sheaths around axons, facilitating a clear distinction between nerve fibers and surrounding tissues. This staining method allows for a detailed examination of motor axon morphology, myelination patterns, and overall structural integrity. In brief, semithin sections of ventral roots at the L5 segment were stained with 1 % toluidine blue and examined under a light microscope, providing a precise histological insight into the organization and health of motor axons.

### 2.6. Statistical analysis

Statistical comparisons between hnRNPA1 transgenic rats and control animals were conducted using an unpaired Student’s *t*-test, which was selected to assess differences between these independent groups. Power calculations were performed based on effect sizes estimated from preliminary histological data. For analyses such as motor neuron quantification, effect sizes ranged from 1.0 to 1.5, supporting the selection of 5–7 animals per group to achieve sufficient statistical power for detecting biologically meaningful differences. The sample sizes employed in this study are therefore justified by these estimations. A p-value of less than 0.05 was defined as the threshold for statistical significance, indicating a low probability that observed differences occurred by chance. While randomization and blinding were not implemented, all experimental procedures, including animal handling and outcome assessments, were conducted under standardized conditions to minimize variability and bias. Power calculations were not conducted a priori, but post hoc analyses confirmed adequate power to detect significant differences in neuron counts.

## 3. Results

### 3.1. Transgenic rats were generated to overexpress hnRNPA1 with an ALS-linked mutation

A mutation in hnRNPA1 has been linked to familial ALS (Kim et al., 2013). To model hnRNPA1-associated pathology, we generated transgenic rats overexpressing hnRNPA1 with the ALS-linked mutation. The presence of D314N mutation was verified by sequencing of PCR products (data not shown). We used rat hnRNPA1 cDNA for overexpression hnRNPA1, as the rat and human proteins are identical at the amino acid level despite differences in their nucleotide sequences. In humans, the ribonucleoprotein hnRNPA1 exhibits two splicing isoforms, with the short isoform being the primary expression (Kim et al., 2013), supporting our selection of the short hnRNPA1 isoform for expression in transgenic rats.

**Fig. 1.**
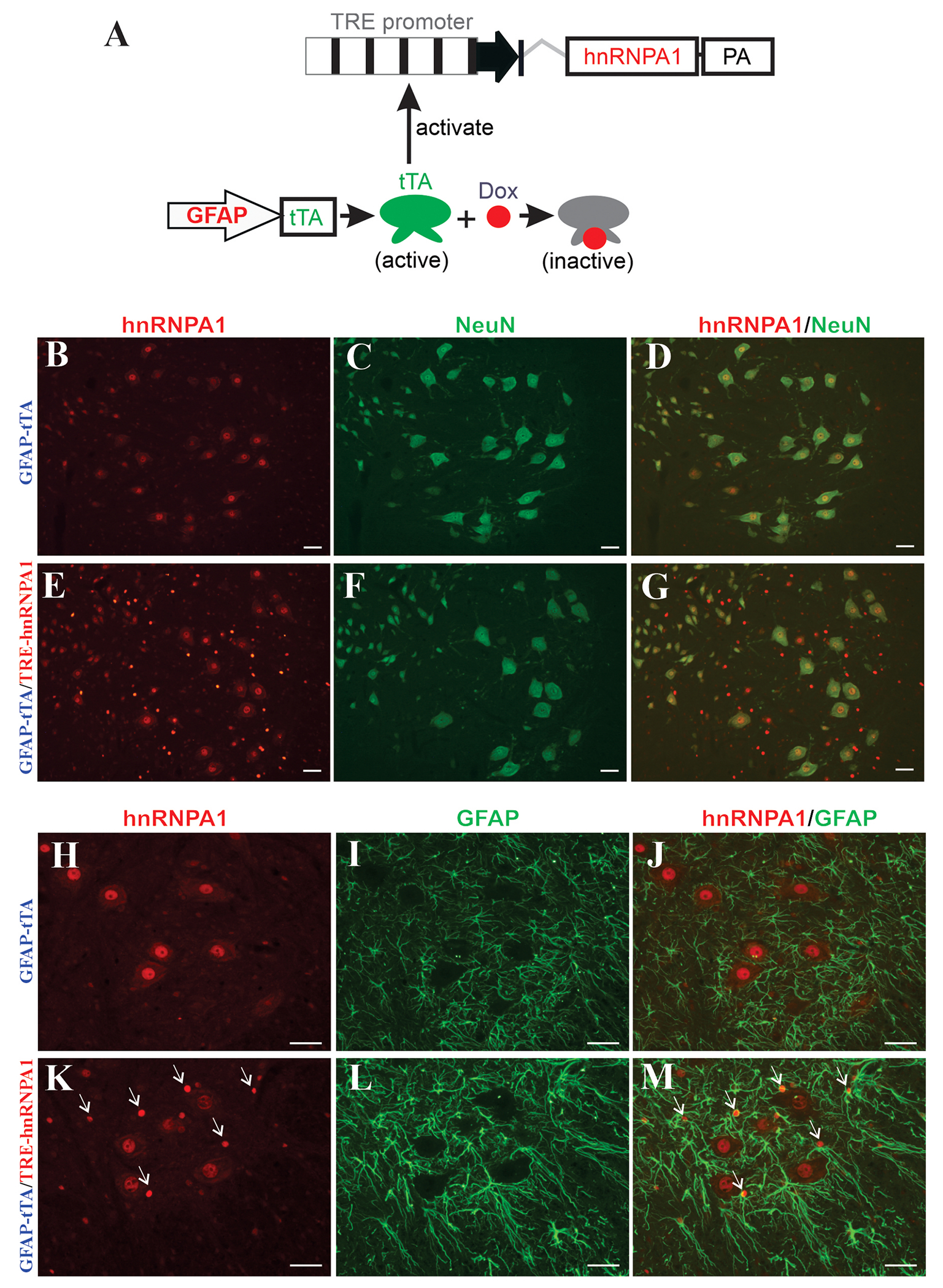
Transgenic rats were generated to overexpress hnRNPA1 in astrocytes. a, A schematic diagram illustrates the structure of hnRNPA1 transgene driven by tetracycline response element (TRE) promoter. These TRE-hnRNPA1 transgenic rats were bred with established GFAP-tTA transgenic rats, resulting in double transgenic offspring that overexpressed hnRNPA1 in astrocytes in the absence of the tTA inactivator Dox. b–g, Immunofluorescence staining revealed heightened hnRNPA1 levels in the spinal cord of GFAP-tTA/TRE-hnRNPA1 double (e–g) transgenic rats as compared to GFAP-tTA single (b–d) transgenic rats. h–m, Double-labeling immunostaining revealed that overexpressed hnRNPA1 was colocalized with the astrocyte marker GFAP in GFAP-tTA/TRE-hnRNPA1 double (k–m), but not in GFAP-tTA single (h–j), transgenic rats. Rats, deprived of Dox at 35 days of age, were examined at 55 days of age. Arrows point to enhanced hnRNPA1 staining in astrocytes (K & M). Scale bars: 50 μm (b–g) and 30 μm (h–m). Representative images were chosen from immunostained sections of three independent animals per genotype.

Using a tetracycline-inducible transgene expression system previously established in the lab, known for its high efficiency in transgene expression (Tong et al., 2013), we employed the TRE promoter to drive the hnRNPA1 transgene (Fig. 1A). A transgenic line exhibiting stable transmission of the TRE-hnRNPA1 transgene was identified (Fig. 1).

### 3.2. Overexpression of pathogenic hnRNPA1 in astrocytes causes progressive paralysis

The amino acid sequence is conserved between human and rat hnRNPA1 proteins, making it unfeasible to generate a species-specific antibody for detecting transgenic hnRNPA1 in transgenic rats. HnRNPA1, a highly conserved ribonucleoprotein with a compact structure, exhibits similarity across species (Kim et al., 2013; Kashima et al., 2007; Liu and Dreyfuss, 1995). To preserve hnRNPA1 functionality in animal models, we opted to express native hnRNPA1 protein without any tags. In the spinal cord of adult rats, hnRNPA1 demonstrated moderate expression in both neuronal and non-neuronal cells (Fig. 1). Expression of hnRNPA1 transgene is governed by the TRE promoter, contingent on the temporal and spatial patterns of GFAP-tTA transgene expression (Fig. 1A). The GFAP-tTA transgenic rat line employed in this study was extensively characterized in a previous study, confirming the specific expression of transgenes in astrocytes (Tong et al., 2013). To express mutant hnRNPA1 specifically in astrocytes, TRE-hnRNPA1 transgenic rats were bred with GFAP-tTA transgenic rats, resulting in double transgenic offspring. Immunofluorescent staining validated the expression of mutant hnRNPA1 in astrocytes (Fig. 1).

**Fig. 2.**
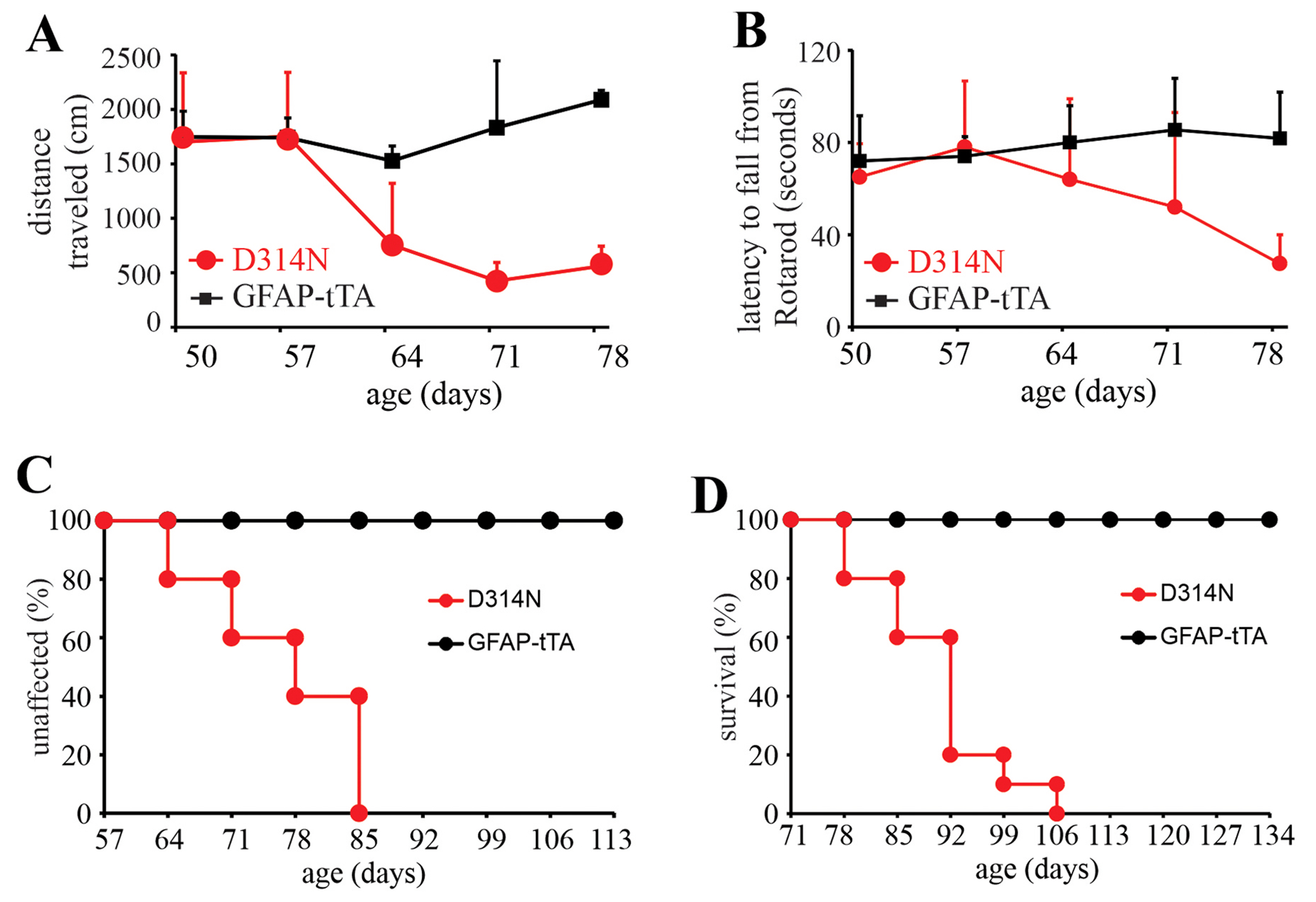
Restricted overexpression of pathogenic hnRNPA1 in astrocytes led to progressive paralysis in transgenic rats. a, Open field assay revealed that mobility was progressively decreased in rats expressing mutant hnRNPA1 (D314N). b, Rotarod test revealed that psychomotor activity was progressively reduced in hnRNPA1 transgenic rats. c-d, Diagrams depict the rates of disease onset and animal survival for mutant (D314N) hnRNPA1 transgenic rats, with GFAP-tTA single transgenic rats serving as normal controls. The median age of disease onset is 76 days (c) and the median survival is 90 days (d). Both GFAP-tTA single and GFAP-tTA/TRE-hnRNPA1 double (D314N) transgenic rats were deprived of Dox at 35 days of age and behaviorally examined from 50 days of age onward (n = 12; equal number of males and females).

Following an established protocol (Tong et al., 2013; Huang et al., 2012), we induced hnRNPA1 transgene activation in postnatal rats by providing breeding females and their offspring with Doxycycline-containing water until the offspring reached 35 days old (Fig. 2). This transgene induction strategy was designed to elicit hnRNPA1-associated phenotypes in adult rats (Fig. 2), facilitating subsequent behavior tests. Overexpression of pathogenic hnRNPA1 in astrocytes led to progressive paralysis in the transgenic rats (Fig. 2). Onset and progression of ALS-like paralysis was discerned through behavioral tests, including Open Field assay and Rotarod test (Fig. 2A–B). In alignment with our findings in TDP-43 transgenic studies (Mitchell et al., 2013; Tsai et al., 2010), our data indicate that the overexpression of pathogenic hnRNPA1 in astrocytes caused progressive paralysis in transgenic rats.

### 3.3. Expression of mutant hnRNPA1 in astrocytes results in a severe loss of motor neurons

**Fig. 3.**
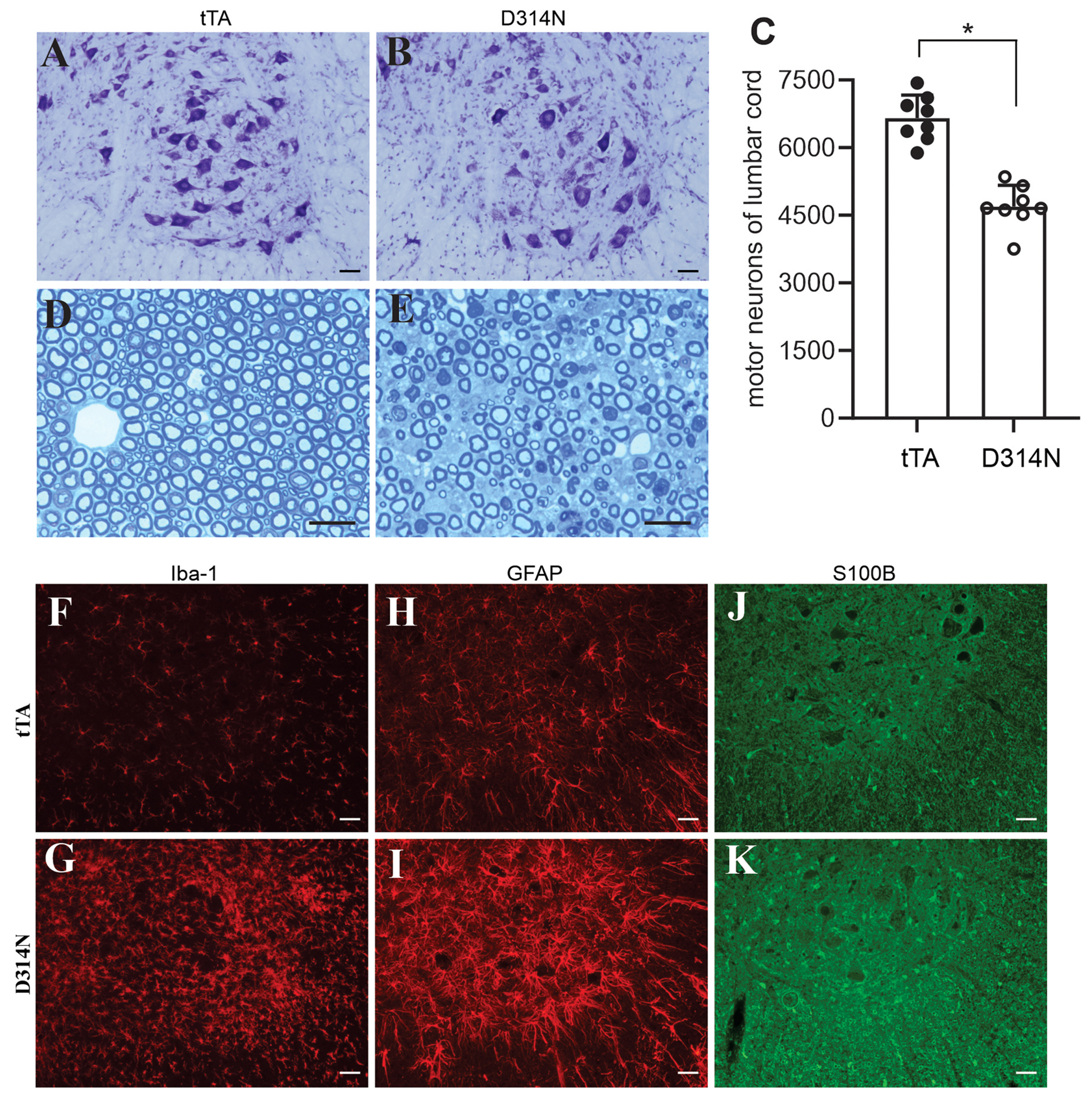
Expression of mutant hnRNPA1 in astrocytes resulted in motor neuron death. a-b, Cresyl violet staining illustrated motor neurons in the ventral horns of rat spinal cords. c, Stereological cell counting quantified motor neurons (diameter > 25 μm) in the L3-L5 lumbar cords of GFAPtTA single (tTA) and GFAP-tTA/TRE-hnRNPA1 double (D314N) transgenic rats. Data are means ± SD (n = 8; equal number of males and females). * *p* < 0.05. d-e, Toluidine blue staining revealed the structures of L5 ventral roots in GFAPtTA single (tTA) and GFAP-tTA/TRE-hnRNPA1^D314N^ double (D314N) transgenic rats. f–k, Immunofluorescence staining displayed microglia (f-g) and astrocytes (h-k) in the ventral horns of rat spinal cords. Mutant hnRNPA1 transgenic rats (D314N) were examined at paralysis stages, and control rats (tTA) were examined at matched ages. Scale bars: 30 μm (d, e) and 50 μm (a-b & f–k). Representative images were chosen from immunostained sections of three independent animals per genotype.

**Fig. 4.**
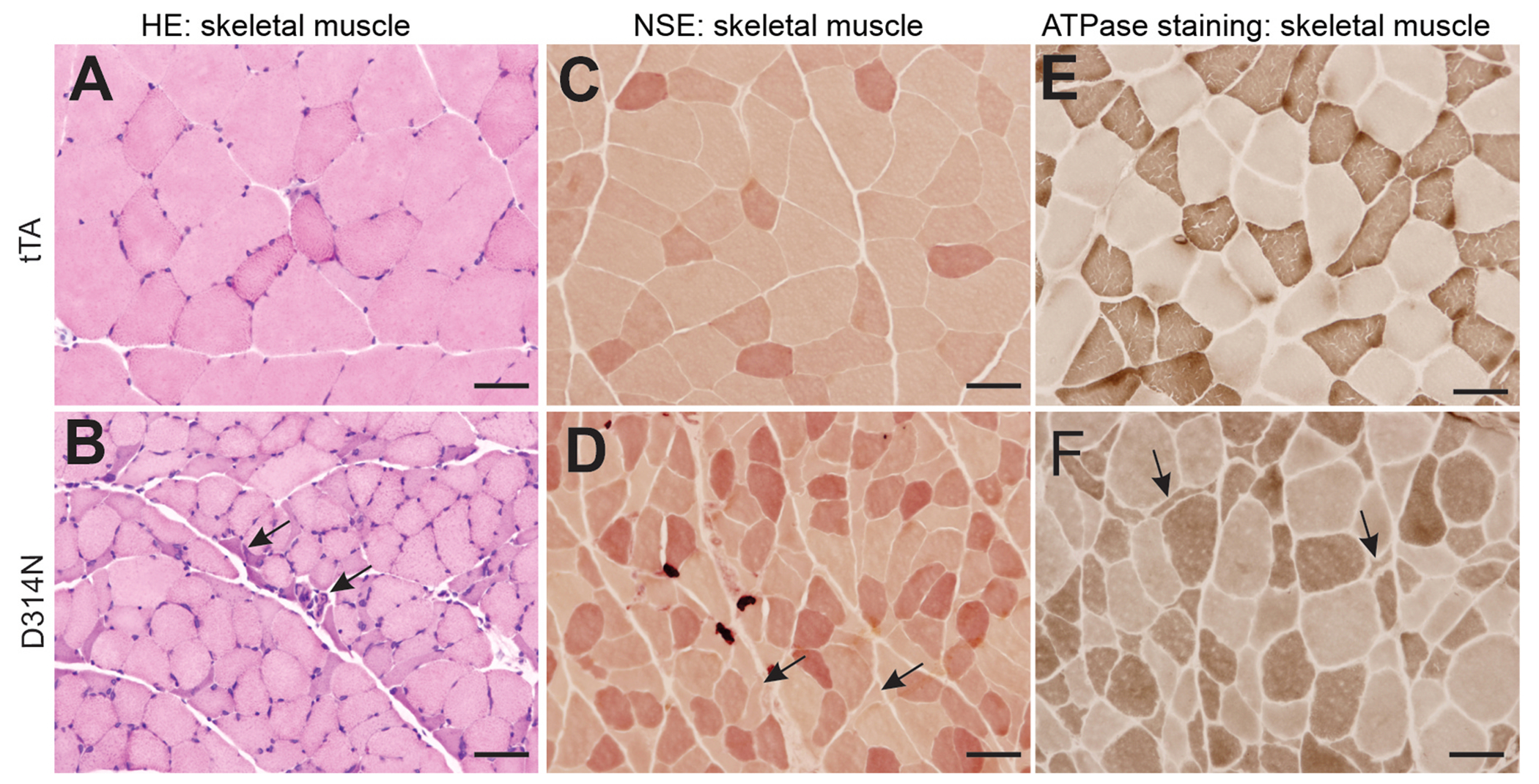
Overexpression of mutant hnRNPA1 in astrocytes led to subsequent muscle atrophy in transgenic rats. a–f, Gastrocnemius muscles were assessed through H&E staining (a-b, HE), histochemistry for nonspecific esterase (c-d, NSE), and ATPase staining (e-f). GFAP-tTA/TRE-hnRNPA1^D314N^ double (D314N) transgenic rats were examined at paralysis stages, while GFAPtTA single (tTA) transgenic rats serving as the control were examined at corresponding ages. Arrows point to atrophied muscle fibers (B, D, & F). Scale bars: 50 μm. Representative images were chosen from immunostained sections of three independent animals per genotype.

The predominant phenotype observed in GFAP-tTA/TRE-hnRNPA1 transgenic rats was paralysis (Fig. 2), likely arising from dysfunction or damage to motor unit. Initially, we assessed mutant hnRNPA1 transgenic rats for motor neuron loss. Cresyl violet staining indicated a reduction in motor neuron density in the spinal cord of mutant hnRNPA1 transgenic rats (Fig. 3A–C). Concurrently, motor axons in the ventral roots exhibited severe damage due to motor neuron death (Fig. 3D–E). Unbiased stereological cell counting corroborated the loss of motor neurons in hnRNPA1 transgenic rats experiencing paralysis (Fig. 3C). In response to motor neuron death, both microglia and astrocytes displayed significant activation, as evidenced by immunofluorescence staining (Fig. 3F–K). As a consequence of motor neuron death, skeletal muscles underwent grouped atrophy, as revealed by H&E staining (Fig. 4A–B), histochemical staining for nonspecific esterase (Fig. 4C–D), and histochemical staining for ATPase (Fig. 4E–F). Our analysis of hnRNPA1 transgenic rats indicated that astrocytes overexpressing mutant hnRNPA1 induced non-cell-autonomous motor neuron death (Fig. 3).

### 3.4. Mutant hnRNPA1 transgenic rats display no detectable brain pathology at paralysis stages

**Fig. 5.**
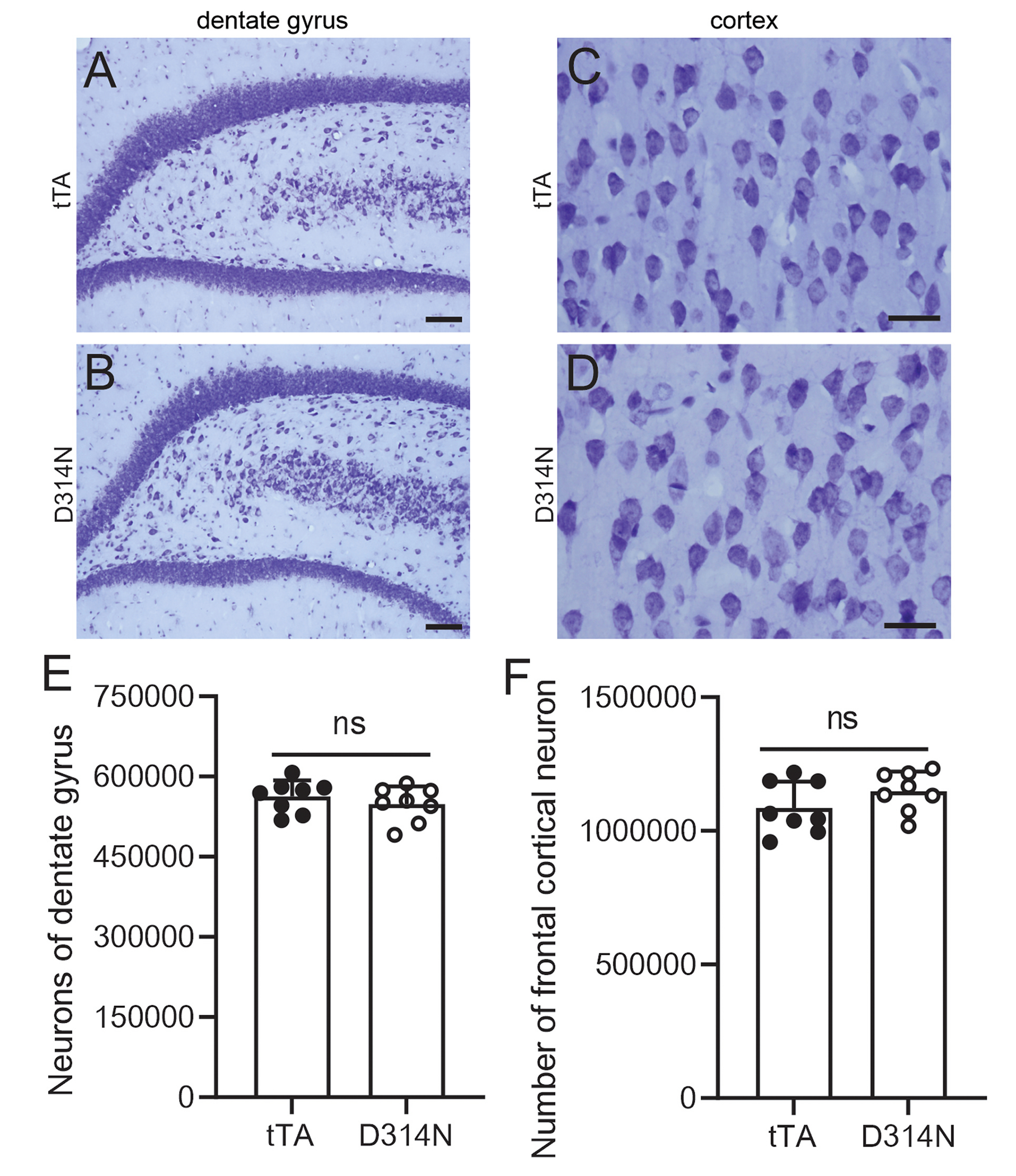
Unbiased cell counting revealed no neuron loss in the brain of mutant hnRNPA1 transgenic rats. a–d, Nissl staining displayed neurons in the dentate gyrus and frontal cortex of rat’s brains. Scale bars: 100 μm (a-b) and 30 μm (c-d). e-f, Stereological cell counting quantified neurons (diameter > 25 μm) in the dentate gyrus and frontal cortex for both GFAPtTA single (tTA) and GFAP-tTA/TRE-hnRNPA1 double (D314N) transgenic rats. There was no significant difference in neuron number between tTA and D314N rats. Data are means ± SD (n = 8; equal number of males and females).

In our transgenic rats expressing mutant hnRNPA1 specifically in astrocytes, we observed that the predominant phenotype was paralysis (Figs. 1–3). Considering the residence of astrocytes in the central nervous system, we opted to investigate the rat’s brain for neuronal loss. Cresyl violet staining indicated no structural alterations in the frontal cortex and hippocampus of mutant hnRNPA1 transgenic rats at paralysis stages (Fig. 5A–D). Using unbiased stereological neuron counting, we estimated the number of neurons in the frontal cortex and the dentate gyrus of transgenic rats. No significant loss of neurons was observed in mutant hnRNPA1 transgenic rats at paralysis stages (Fig. 5E–F). These findings suggest that spinal cord motor neurons were primarily affected by overexpression of pathogenic hnRNPA1 in astrocytes.

## 4. Discussion

Pathogenic mutation in hnRNPA1 segregates with ALS in affected families (Kim et al., 2013). Our research in transgenic rats demonstrates that the overexpression of mutant hnRNPA1 induces progressive paralysis. This overexpression results in the loss of motor neurons and the denervation atrophy of skeletal muscles. Specifically, the D314N substitution in hnRNPA1, identified in ALS patients (Kim et al., 2013), was scrutinized in our transgenic studies. Our findings in hnRNPA1 transgenic rats support the notion that the hnRNPA1 mutation is causative of motor neuron degeneration. Notably, in our rat model where mutant hnRNPA1 was selectively expressed in astrocytes, motor neurons underwent non-cell-autonomous death.

It is noteworthy that the presence of a disease-causative factor in both neurons and glial cells may not be imperative to initiate the disease process. Instead, the presence of this factor in a single cell type, such as astrocytes, seems sufficient to instigate neurodegeneration. Our experiment substantiates that the constrained expression of mutant hnRNPA1 in astrocytes results in motor neuron death and the manifestation of ALS-like characteristics. Both hnRNPA1 and TDP-43 belong to the ribonucleoprotein family and are associated with motor neuron degeneration in ALS (Sreedharan et al., 2008; Kim et al., 2013; Kabashi et al., 2008). The restricted expression of mutant TDP-43 in astrocytes induces non-cell-autonomous motor neuron death, with astrocytic neurotoxicity likely stemming from a loss of neuroprotective functions and a gain of neurotoxic properties in aberrant astrocytes (Tong et al., 2013; Huang et al., 2014). Astrocytes, essential support cells in the central nervous system, contribute to non-cell-autonomous neuron death possibly through various mechanisms. Dysfunction in these glial cells compromises neuronal health by impairing their supportive functions (Gao et al., 2022; Gutbier et al., 2018; Ren et al., 2020). Activated astrocytes release pro-inflammatory molecules (Lu et al., 2023; Matoba et al., 2022; Ng and Ng, 2022; Quan et al., 2023; Torazza et al., 2023), fostering a toxic environment detrimental to neighboring neurons. Additionally, failure to properly regulate extracellular glutamate can induce excitotoxicity (Torazza et al., 2023; Andersen and Schousboe, 2023; Andersen and Schousboe, 2023; Provenzano et al., 2023; Schmitt et al., 2023), further leading to neuronal damage. Altered astrocyte-neuron signaling disrupts the release of crucial neurotrophic factors (Chang et al., 2023; Park et al., 2023), affecting overall neuron viability. Metabolic dysfunction in astrocytes compromises energy supply to neurons (Brazhe et al., 2023; Nunes et al., 2023; Rabah et al., 2023), contributing to neuron damage. Dysregulated gliotransmitter release and the generation of reactive oxygen species by dysfunctional astrocytes can disrupt synaptic function and cause oxidative stress (Dey et al., 2023; Gil-Jaramillo et al., 2023), respectively, both culminating in neuronal damage. Moreover, astrocyte-mediated breakdown of the blood-brain barrier allows the entry of harmful substances (Zhang et al., 2023; Zhou et al., 2023), exacerbating the overall impact on neuronal health. While the current study primarily focused on astrocytes, other glial cell types, such as microglia, also play critical roles in non-cell-autonomous neurodegeneration. Notably, microglial activation was observed in proximity to degenerating motor neurons in transgenic rats expressing pathogenic hnRNPA1 specifically in astrocytes. These findings suggest that dynamic interactions between glial cell types may contribute to astrocyte-mediated non-cell-autonomous neurodegeneration. Given the essential role of astrocytes in maintaining the structure and function of central nervous system (Khakh and Sofroniew, 2015), substantial changes in the expression of critical genes (e.g., TDP-43 and hnRNPA1) likely transform healthy astrocytes into an aberrant status (Tong et al., 2013), leading to non-cell-autonomous neuron death.

This study was deliberately developed as an *in vivo* proof-of-concept model to evaluate whether astrocytic expression of pathogenic hnRNPA1 alone is sufficient to trigger non-cell-autonomous neurodegeneration. While our results support this hypothesis, the precise mechanisms by which astrocytes mediate motor neuron toxicity remain to be elucidated. While our data provide strong evidence that astrocyte-specific expression of mutant hnRNPA1 is sufficient to induce motor neuron degeneration, further validation is required to establish the mutation-specific effects of hnRNPA1. This will necessitate direct comparisons between astrocyte-targeted transgenic lines expressing either wild-type or mutant hnRNPA1. These findings provide a foundational framework for future investigations aimed at delineating astrocytic stress responses, identifying secreted neurotoxic factors, and characterizing the glial-neuronal interactions that contribute to hnRNPA1-associated motor neuron pathology.

Both human and rat hnRNPA1 proteins have the identical sequence of amino acids, making the generation of a species-specific antibody for detecting transgenic hnRNPA1 unlikely. In our transgenic rats, hnRNPA1 transgene was driven by the TRE promoter, and its expression relied on the transcriptional activity of tTA. The expression of the hnRNPA1 transgene is under the control of the TRE promoter, dependent on the temporal and spatial patterns of tTA transgene expression. In a previous study, we extensively characterized the GFAP-tTA transgenic rat line, known for selectively expressing transgenes in astrocytes (Tong et al., 2013). However, our current methodology does not allow us to unequivocally differentiate the transgenic hnRNPA1 from its endogenous counterpart within astrocytes. Our findings indicate that motor neurons are particularly susceptible in the context of hnRNPA1 overexpression. However, given the considerably lower basal expression of hnRNPA1 in astrocytes relative to motor neurons (Fig. 1), it is conceivable that overexpression of even the wild-type protein could elicit a similar phenotype. Thus, while the mutation may intensify pathogenic processes, the current data do not unequivocally distinguish between the effects of aberrant protein expression and mutation-specific mechanisms. Further investigations, directly comparing the consequences of overexpressing wild-type and mutant hnRNPA1 in astrocytes, are warranted to definitively delineate these contributions.

Our findings indicate that when the ALS-linked gene hnRNPA1 is overexpressed in astrocytes, it preferentially impacts motor neurons in a non-cell-autonomous manner. Similar to other ALS-associated ribonucleoproteins such as TDP-43 and FUS, hnRNPA1 is ubiquitously present in various cell types, including astrocytes. While exclusively expressing pathogenic hnRNPA1 in astrocytes may not fully replicate the actual hnRNPA1 expression patterns in patients, our study is specifically designed to investigate the cellular mechanisms of astrocyte-mediated non-cell-autonomous motor neuron death in the disease. It’s crucial to emphasize that comprehending these mechanisms is essential for the development of effective therapeutic strategies. In the context of therapeutic approaches, targeting astrocytes or astrocyte-specific pathways becomes paramount for maximizing therapeutic efficacy.

## Data Availability

Data will be made available on request.

## References

[R1] Andersen JV, Schousboe A, 2023. Glial glutamine homeostasis in health and disease. Neurochem. Res 48, 1100–1128. 36322369 10.1007/s11064-022-03771-1

[R2] Andersen JV, Schousboe A, 2023. Milestone review: metabolic dynamics of glutamate and GABA mediated neurotransmission - the essential roles of astrocytes.J. Neurochem 166, 109–137. 36919769 10.1111/jnc.15811

[R3] Beal MF, 2001. Mitochondria and oxidative damage in amyotrophic lateral sclerosis. Funct. Neurol 16, 161–169. 11996512

[R4] Bittigau P, Ikonomidou C, 1997. Glutamate in neurologic diseases. J. Child Neurol 12, 471–485. 9430311 10.1177/088307389701200802

[R5] Brazhe A, Verisokin A, Verveyko D, Postnov D, 2023. Astrocytes: new evidence, new models, new roles. Biophys. Rev 15, 1303–1333. 37975000 10.1007/s12551-023-01145-7 PMC10643736

[R6] Brown RH Jr., Robberecht W, 2001. Amyotrophic lateral sclerosis: pathogenesis. Semin. Neurol 21, 131–139. 11442322 10.1055/s-2001-15260

[R7] Chang J, Qian Z, Wang B, Cao J, Zhang S, Jiang F, , 2023. Transplantation of A2 type astrocytes promotes neural repair and remyelination after spinal cord injury (CCS). Cell Commun. Signal 21, 37. 36797790 10.1186/s12964-022-01036-6 PMC9936716

[R8] Cleveland DW, Rothstein JD, 2001. From charcot to lou gehrig: deciphering selective motor neuron death in ALS. Nat. Rev. Neurosci 2, 806–819. 11715057 10.1038/35097565

[R9] Custer SK, Garden GA, Gill N, Rueb U, Libby RT, Schultz C, , 2006. Bergmann glia expression of polyglutamine-expanded ataxin-7 produces neurodegeneration by impairing glutamate transport. Nat. Neurosci 9, 1302–1311. 16936724 10.1038/nn1750

[R10] Deng HX, Hentati A, Tainer JA, Iqbal Z, Cayabyab A, Hung WY, , 1993. Amyotrophic lateral sclerosis and structural defects in Cu,Zn superoxide dismutase. Science 261, 1047–1051. 8351519 10.1126/science.8351519

[R11] Dey A, Pramanik PK, Dwivedi SKD, Neizer-Ashun F, Kiss T, Ganguly A, , 2023. A role for the cystathionine-β-synthase /H(2)S axis in astrocyte dysfunction in the aging brain. Redox Biol. 68, 102958. 37948927 10.1016/j.redox.2023.102958 PMC10663824

[R12] Gagné M, Deshaies JE, Sidibé H, Benchaar Y, Arbour D, Dubinski A, , 2021. hnRNP A1B, a splice variant of HNRNPA1, is spatially and temporally regulated. Front. Neurosci 15, 724307. 34630013 10.3389/fnins.2021.724307 PMC8498194

[R13] Gao R, Chen Z, Wu Y, Chen R, Zheng W, Qi L, , 2022. SIRT3 alleviates mitochondrial dysfunction induced by recurrent low glucose and improves the supportive function of astrocytes to neurons. Free Radic. Biol. Med 193, 405–420. 36306990 10.1016/j.freeradbiomed.2022.10.313

[R14] Gendron TF, Belzil VV, Zhang YJ, Petrucelli L, 2014. Mechanisms of toxicity in C9FTLD/ALS. Acta Neuropathol. 127, 359–376. 24394885 10.1007/s00401-013-1237-z PMC4002260

[R15] Gil-Jaramillo N, Aristizábal-Pachón AF, Luque Aleman MA, González Gómez V, Escobar Hurtado HD, Girón Pinto LC, , 2023. Competing endogenous RNAs in human astrocytes: crosstalk and interacting networks in response to lipotoxicity. Front. Neurosci 17, 1195840. 38027526 10.3389/fnins.2023.1195840 PMC10679742

[R16] Gurney ME, Pu H, Chiu AY, Dal Canto MC, Polchow CY, Alexander DD, , 1994. Motor neuron degeneration in mice that express a human Cu,Zn superoxide dismutase mutation. Science 264, 1772–1775. 8209258 10.1126/science.8209258

[R17] Gutbier S, Spreng AS, Delp J, Schildknecht S, Karreman C, Suciu I, , 2018. Prevention of neuronal apoptosis by astrocytes through thiol-mediated stress response modulation and accelerated recovery from proteotoxic stress. Cell Death Differ. 25, 2101–2117. 30390092 10.1038/s41418-018-0229-x PMC6261954

[R18] Hay DC, Kemp GD, Dargemont C, Hay RT, 2001. Interaction between hnRNPA1 and IkappaBalpha is required for maximal activation of NF-kappaB-dependent transcription. Mol. Cell Biol 21, 3482–3490. 11313474 10.1128/MCB.21.10.3482-3490.2001 PMC100270

[R19] Huang C, Huang B, Bi F, Yan LH, Tong J, Huang J, , 2014. Profiling the genes affected by pathogenic TDP-43 in astrocytes. J. Neurochem 129, 932–939. 24447103 10.1111/jnc.12660 PMC4066372

[R20] Huang C, Tong J, Bi F, Zhou H, Xia XG, 2012. Mutant TDP-43 in motor neurons promotes the onset and progression of ALS in rats. J. Clin. Invest 122, 107–118. 22156203 10.1172/JCI59130 PMC3248298

[R21] Ikonomidou C, Qin Qin Y, Labruyere J, Olney JW, 1996. Motor neuron degeneration induced by excitotoxin agonists has features in common with those seen in the SOD-1 transgenic mouse model of amyotrophic lateral sclerosis.J. Neuropathol. Exp. Neurol 55, 211–224. 8786380 10.1097/00005072-199602000-00010

[R22] Ilieva H, Polymenidou M, Cleveland DW, 2009. Non-cell autonomous toxicity in neurodegenerative disorders: ALS and beyond. J. Cell Biol 187, 761–772. 19951898 10.1083/jcb.200908164 PMC2806318

[R23] Jean-Philippe J, Paz S, Caputi M, 2013. hnRNP A1: the Swiss army knife of gene expression. Int. J. Mol. Sci 14, 18999–19024. 24065100 10.3390/ijms140918999 PMC3794818

[R24] Kabashi E, Valdmanis PN, Dion P, Spiegelman D, McConkey BJ, Velde CV, , 2008. TARDBP mutations in individuals with sporadic and familial amyotrophic lateral sclerosis. Nat. Genet 40, 572–574. 18372902 10.1038/ng.132

[R25] Kabashi E, Valdmanis PN, Dion P, Spiegelman D, McConkey BJ, Vande Velde C, , 2008. TARDBP mutations in individuals with sporadic and familial amyotrophic lateral sclerosis. Nat. Genet 40, 572–574. 18372902 10.1038/ng.132

[R26] Kashima T, Rao N, David CJ, Manley JL, 2007. hnRNP A1 functions with specificity in repression of SMN2 exon 7 splicing. Hum. Mol. Genet 16, 3149–3159. 17884807 10.1093/hmg/ddm276

[R27] Khakh BS, Sofroniew MV, 2015. Diversity of astrocyte functions and phenotypes in neural circuits. Nat. Neurosci 18, 942–952. 26108722 10.1038/nn.4043 PMC5258184

[R28] Kim HJ, Kim NC, Wang YD, Scarborough EA, Moore J, Diaz Z, , 2013. Mutations in prion-like domains in hnRNPA2B1 and hnRNPA1 cause multisystem proteinopathy and ALS. Nature 495, 467–473. 23455423 10.1038/nature11922 PMC3756911

[R29] Kim S, Park GH, Paik WK, 1998. Recent advances in protein methylation: enzymatic methylation of nucleic acid binding proteins. Amino Acids 15, 291–306. 9891755 10.1007/BF01320895

[R30] Ko DC, Milenkovic L, Beier SM, Manuel H, Buchanan J, Scott MP, 2005. Cell-autonomous death of cerebellar purkinje neurons with autophagy in Niemann-Pick type c disease. PLoS Genet. 1, 81–95. 16103921 10.1371/journal.pgen.0010007 PMC1183526

[R31] Kwiatkowski TJ, Bosco DA Jr., Leclerc AL, Tamrazian E, Vanderburg CR, Russ C, , 2009. Mutations in the FUS/TLS gene on chromosome 16 cause familial amyotrophic lateral sclerosis. Science 323, 1205–1208. 19251627 10.1126/science.1166066

[R32] Lian C, Zhang C, Tian P, Tan Q, Wei Y, Wang Z, , 2024. Epigenetic reader ZMYND11 noncanonical function restricts HNRNPA1-mediated stress granule formation and oncogenic activity. Signal Transduct. Target. Ther 9, 258. 39341825 10.1038/s41392-024-01961-7 PMC11438962

[R33] Liu Q, Dreyfuss G, 1995. In vivo and in vitro arginine methylation of RNA-binding proteins. Mol. Cell Biol 15, 2800–2808. 7739561 10.1128/mcb.15.5.2800 PMC230511

[R34] Lu W, Chen Z, Wen J, 2023. Flavonoids and ischemic stroke-induced neuroinflammation: focus on the glial cells. Biomed. Pharmacother. = Biomed. Pharmacother 170, 115847. 38016362 10.1016/j.biopha.2023.115847

[R35] Matoba K, Dohi E, Choi EY, Kano SI, 2022. Glutathione S-transferases control astrocyte activation and neuronal health during neuroinflammation. Front. Mol. Biosci 9, 1080140. 36685285 10.3389/fmolb.2022.1080140 PMC9853189

[R36] Menzies FM, Ince PG, Shaw PJ, 2002. Mitochondrial involvement in amyotrophic lateral sclerosis. Neurochem. Int 40, 543–551. 11850111 10.1016/s0197-0186(01)00125-5

[R37] Mitchell JC, McGoldrick P, Vance C, Hortobagyi T, Sreedharan J, Rogelj B, , 2013. Overexpression of human wild-type FUS causes progressive motor neuron degeneration in an age- and dose-dependent fashion. Acta Neuropathol. 125, 273–288. 22961620 10.1007/s00401-012-1043-z PMC3549237

[R38] Ng W, Ng SY, 2022. Remodeling of astrocyte secretome in amyotrophic lateral sclerosis: uncovering novel targets to combat astrocyte-mediated toxicity. Transl. Neurodegener 11, 54. 36567359 10.1186/s40035-022-00332-y PMC9791755

[R39] Nunes MJ, Carvalho AN, Sá-Lemos C, Colaço M, Cervenka I, Ciraci V, , 2023. Sustained PGC-1α2 or PGC-1α3 expression induces astrocyte dysfunction and degeneration. Eur. J. Cell Biol 103, 151377. 38006841 10.1016/j.ejcb.2023.151377

[R40] Park JE, Leem YH, Park JS, Kim SE, Kim HS, 2023. Astrocytic Nrf2 mediates the neuroprotective and Anti-Inflammatory effects of nootkatone in an MPTP-Induced parkinson’s disease mouse model. Antioxidants 12. 10.3390/antiox12111999 PMC10669233 38001852

[R41] Pastor T, Pagani F, 2011. Interaction of hnRNPA1/A2 and DAZAP1 with an Alu-derived intronic splicing enhancer regulates ATM aberrant splicing. PLoS One 6, e23349. 21858080 10.1371/journal.pone.0023349 PMC3152568

[R42] Pramatarova A, Laganiere J, Roussel J, Brisebois K, Rouleau GA, 2001. Neuron-specific expression of mutant superoxide dismutase 1 in transgenic mice does not lead to motor impairment. J. Neurosci 21, 3369–3374. 11331366 10.1523/JNEUROSCI.21-10-03369.2001 PMC6762496

[R43] Provenzano F, Torazza C, Bonifacino T, Bonanno G, Milanese M, 2023. The key role of astrocytes in amyotrophic lateral sclerosis and their commitment to glutamate excitotoxicity. Int. J. Mol. Sci 24. 10.3390/ijms242015430 PMC10607805 37895110

[R44] Quan W, Xu CS, Li XC, Yang C, Lan T, Wang MY, , 2023. Telmisartan inhibits microglia-induced neurotoxic A1 astrocyte conversion via PPARγ-mediated NF-κB/p65 degradation. Int. Immunopharmacol 123, 110761. 37544025 10.1016/j.intimp.2023.110761

[R45] Rabah Y, Francés R, Minatchy J, Guédon L, Desnous C, Plaçais PY, , 2023. Glycolysis-derived alanine from glia fuels neuronal mitochondria for memory in drosophila. Nat. Metab 5, 2002–2019. 37932430 10.1038/s42255-023-00910-y PMC10663161

[R46] Ren YZ, Zhang BZ, Zhao XJ, Zhang ZY, 2020. Resolvin D1 ameliorates cognitive impairment following traumatic brain injury via protecting astrocytic mitochondria. J. Neurochem 154, 530–546. 31951012 10.1111/jnc.14962

[R47] Rosen DR, Siddique T, Patterson D, Figlewicz DA, Sapp P, Hentati A, , 1993. Mutations in Cu/Zn superoxide dismutase gene are associated with familial amyotrophic lateral sclerosis. Nature 362, 59–62. 8446170 10.1038/362059a0

[R48] Rothstein JD, 1996. Excitotoxicity hypothesis. Neurology 47, S19–S25. 8858047 10.1212/wnl.47.4_suppl_2.19s

[R49] Rutherford NJ, Zhang YJ, Baker M, Gass JM, Finch NA, Xu YF, , 2008. Novel mutations in TARDBP (TDP-43) in patients with familial amyotrophic lateral sclerosis. PLoS Genet. 4, e1000193. 18802454 10.1371/journal.pgen.1000193 PMC2527686

[R50] Schmitt LI, David C, Steffen R, Hezel S, Roos A, Schara-Schmidt U, , 2023. Spinal astrocyte dysfunction drives motor neuron loss in late-onset spinal muscular atrophy. Acta Neuropathol. 145, 611–635. 36930296 10.1007/s00401-023-02554-4 PMC10119066

[R51] Shen EC, Henry MF, Weiss VH, Valentini SR, Silver PA, Lee MS, 1998. Arginine methylation facilitates the nuclear export of hnRNP proteins. Genes Dev. 12, 679–691. 9499403 10.1101/gad.12.5.679 PMC316575

[R52] Siddique T, Lalani I, 2002. Genetic aspects of amyotrophic lateral sclerosis. Adv. Neurol 88, 21–32. 11908227

[R53] Sreedharan J, Blair IP, Tripathi VB, Hu X, Vance C, Rogelj B, , 2008. TDP-43 mutations in familial and sporadic amyotrophic lateral sclerosis. Science 319, 1668–1672. 18309045 10.1126/science.1154584 PMC7116650

[R54] Tong J, Huang C, Bi F, Wu Q, Huang B, Zhou H, 2012. XBP1 depletion precedes ubiquitin aggregation and Golgi fragmentation in TDP-43 transgenic rats.J. Neurochem 123, 406–416. 22970712 10.1111/jnc.12014 PMC3534861

[R55] Tong J, Huang C, Bi F, Wu Q, Huang B, Liu X, , 2013. Expression of ALS-linked TDP-43 mutant in astrocytes causes non-cell-autonomous motor neuron death in rats. EMBO J. 32, 1917–1926. 23714777 10.1038/emboj.2013.122 PMC3981181

[R56] Torazza C, Provenzano F, Gallia E, Cerminara M, Balbi M, Bonifacino T, , 2023. Genetic downregulation of the metabotropic glutamate receptor type 5 dampens the reactive and neurotoxic phenotype of adult ALS astrocytes. Cells 12. 10.3390/cells12151952 PMC10416852 37566031

[R57] Tsai KJ, Yang CH, Fang YH, Cho KH, Chien WL, Wang WT, , 2010. Elevated expression of TDP-43 in the forebrain of mice is sufficient to cause neurological and pathological phenotypes mimicking FTLD-U. J. Exp. Med 207, 1661–1673. 20660618 10.1084/jem.20092164 PMC2916125

[R58] Van Deerlin VM, Leverenz JB, Bekris LM, Bird TD, Yuan W, Elman LB, , 2008. TARDBP mutations in amyotrophic lateral sclerosis with TDP-43 neuropathology: a genetic and histopathological analysis. Lancet Neurol. 7, 409–416. 18396105 10.1016/S1474-4422(08)70071-1 PMC3546119

[R59] Vance Rogelj, C., Hortobagyi B, De Vos T, Nishimura AL KJ, Sreedharan J, , 2009. Mutations in FUS, an RNA processing protein, cause familial amyotrophic lateral sclerosis type 6. Science 323, 1208–1211. 19251628 10.1126/science.1165942 PMC4516382

[R60] Williamson TL, Cleveland DW, 1999. Slowing of axonal transport is a very early event in the toxicity of ALS-linked SOD1 mutants to motor neurons. Nat. Neurosci 2, 50–56. 10195180 10.1038/4553

[R61] Yamanaka K, Chun SJ, Boillee S, Fujimori-Tonou N, Yamashita H, Gutmann DH, , 2008. Astrocytes as determinants of disease progression in inherited amyotrophic lateral sclerosis. Nat. Neurosci 11 (3), 251. 18246065 10.1038/nn2047 PMC3137510

[R62] Zhang S, Li M, Qiu Y, Wu J, Xu X, Ma Q, , 2023. Enhanced VEGF secretion and blood-brain barrier disruption: radiation-mediated inhibition of astrocyte autophagy via PI3K-AKT pathway activation. Glia. 10.1002/glia.24491 38009296

[R63] Zhang B, Tu P, Abtahian F, Trojanowski JQ, Lee VM, 1997. Neurofilaments and orthograde transport are reduced in ventral root axons of transgenic mice that express human SOD1 with a G93A mutation. J. Cell Biol 139, 1307–1315. 9382875 10.1083/jcb.139.5.1307 PMC2140205

[R64] Zhou S, Liu C, Wang J, Ye J, Lian Q, Gan L, , 2023. CCL5 mediated astrocyte-T cell interaction disrupts blood-brain barrier in mice after hemorrhagic stroke. J. Cereb. Blood Flow Metab 271678x231214838. 10.1177/0271678X231214838 PMC10870968 37974301

